# Remarks on the External Application of Opium in Cholera

**Published:** 1832-05

**Authors:** 


### CHOLERA.

### *Remarks on the External Application of Opium in Cholera.* By Dr. Bow.

Without attempting even to guess at the mode by which nervous influence is reproduced after expenditure, it is certain that there is no reservoir of dormant influence, from which an organ can be supplied when its function is immoderately exercised: it is supplied, then, at the expense of other organs, whose functions become less active or impaired in consequence; or, if the function of any organ be exercised less actively than usual, then other organs receive an increased supply of nervous influence, and their functions therefore become proportionably more active. The above is meant as introductory to a few remarks on cholera.

In my work on Fever, I profess to be one who believes that the office of the great sympathetic nerve is to effect involuntary motion, and the chemical processes which go on in the system; and I believe with many, and in particular with Mr. G. H. Bell, that to a failure in power in the sympathetic system of nerves "is to be ascribed the disease which has obtained the name of Cholera Asphyxia," and also with him, that "the great object in the cure of the disease is to stimulate the sympathetic system of nerves." But there is only one way by which this system of nerves can effectually be stimulated: of this hereafter. If the functions of the sympathetic nerve be suspended or less actively exercised, where is the proof that the functions of other organs are being exercised more effectually? It is to be found in the fact that the mental faculties remain unimpaired, although black blood is circulating, or perhaps rather stagnating, in the brain. It is to be found in the fact that the skin and stomach acquire a morbid sensibility, sometimes to a painful degree; and in the tonic spasms of the muscles. In a healthy state of the body, we may divert nervous influence from the sympathetic system to the surface of the body, by stimulating the extremities of the sentient nerves by blisters, sinapisms, and other irritating applications; but these are applied, in the disease before us, with the view of producing an opposite effect! Diminish the sensibility of the surface rather than increase it, and we shall then arrive at the true mode of stimulating the sympathetic system. Wash the patient with laudanum, and observe the effect.

Before the cholera appeared in Sunderland, I attended a case of common cholera; for, although it was the worst case I had till then seen, I did not think of discovering any resemblance to the Asiatic. All the common remedies failed to check the vomiting, purging, and cramps; but I at once succeeded with the strong opiate liniment, the formula for which I subjoin.*

A report of this case I forwarded to one of the medical gentlemen sent by government to Sunderland; but, as he took no notice of my letter, I presume he did not receive it, although the dead-letter office has not noticed it either.

The following is a case in which the good effects almost immediately followed the application, and the only other where I have had an opportunity of trying the plan fairly.

Mrs. D., aged twenty-six, the mother of three children, (the youngest sixteen months old, still at the breast,) was attacked, at three in the morning, with vomiting and purging. I saw her at five: she then complained of great thirst, great weakness, and severe cramp in the great toes; there was lividity under the eyes, and coldness of the feet and hands, although she did not complain of it; pulse 104, and feeble; the tongue appeared bloodless, but not very cold. I saw neither the dejections nor the matter vomited.—Calomel, opium, &c.

At half-past eight o'clock, the symptoms were alarming: the cramps had extended to the knees; she could not move out of bed, and there the stools were passed; the medicine was rejected from the stomach, and the matter vomited had the appearance of thin gruel. The dejections I did not see,

* ℞. Opii, ʒj.; Liniment. Camph. c. ℥j. Digere per dies aliquot et effunde linimentum.

but was told afterwards that there appeared nothing but water. The smell of the bed was exactly that which I perceived at Newburn. The pulse was barely perceptible; the lividity under the eyes greatly increased, and the thirst intolerable; the voice weak.—I ordered rather more than half an ounce of the liniment to be rubbed on the bowels and spine; two grains of calomel.

At ten o'clock, I found her lying on her side, with her knees drawn up: she had neither vomited nor purged since the liniment was applied; the tongue had lost its paleness; colour had returned to the cheeks, and there was a glow of heat all over the body. She said she was perfectly relieved, excepting that, when she moved, the cramp returned to the toes.

At eleven o'clock, I found her in a sound sleep: whilst I endeavoured to feel the pulse, she awoke, and said she was perfectly comfortable and happy. Next day, she was able to attend to the business of the house.

When at Newcastle, in January last, I mentioned to Mr. Howey what I conceived to be the nature of the disease, and also the benefit which I expected would result from opiate frictions: upon which he noticed a case where the cure was attributed to the accidental application of two ounces of laudanum. Mr. H. did not himself attend the patient, but he was kind enough to procure for me the following, which is an extract from his letter:

"The case in which the two ounces of laudanum were rubbed upon the bowels, was that of a man named Killbreath, at Dent's Hole, and occurred before I came to this neighbourhood. It appears that Mr. F. found him in the evening, rapidly approaching the stage of collapse; the pulse was extremely weak and thready; hands cold, and rather blue; severe cramps in the legs, with vomiting and purging, the fluid evacuated resembling milk and water. About three pints of warm water were immediately given, followed by an enema of gruel, containing Spt. Ammoniæ. The bowels having been completely emptied by these means, a draught containing Tinct. Opii, gtt. xxiv., was given, and half an ounce directed to be rubbed upon the abdomen; but, by mistake, two ounces were used in that manner. Next morning the man had completely rallied, having passed a comfortable night, without any recurrence of the vomiting."

Dr. Craigie says, that in the choleric collapse the heart's action is certainly augmented in force, as if to overcome an obstacle; and if it succeeds in this, the disease is cured. I have no doubt but that the heart's action is augmented in force for some time after collapse takes place, compared to what its force was in the previous stage; and I have as little doubt but that we have it in our power, in the majority of cases, to enable it to overcome the obstacle, by a plentiful application of opium, belladonna, or the like, externally.

*Alnwick; April* 12, 1832.

