# Erratum

**Published:** 1832-06

**Authors:** 


ERRATUM in the last Number of our Journal.—In the foot note, page 356, for Opium ℥i. read ʒi.

